# Palliative Care in the Global Setting: ASCO Resource-Stratified Practice Guideline

**DOI:** 10.1200/JGO.18.00026

**Published:** 2018-05-08

**Authors:** Hibah Osman, Sudip Shrestha, Sarah Temin, Zipporah V. Ali, Rumalie A. Corvera, Henry D. Ddungu, Liliana De Lima, Maria Del Pilar Estevez-Diz, Frank D. Ferris, Nahla Gafer, Harmala K. Gupta, Susan Horton, Graciela Jacob, Ruinuo Jia, Frank L. Lu, Daniela Mosoiu, Christina Puchalski, Carole Seigel, Olaitan Soyannwo, James F. Cleary

**Affiliations:** **Hibah Osman**, Balsam–Lebanese Center for Palliative Care, American University of Beirut Medical Center, Beirut, Lebanon; **Sudip Shrestha**, Nepal Cancer Hospital and Research Center, Lalitpur Submetropolis, Nepal; **Sarah Temin**, ASCO, Alexandria, VA; **Zipporah V. Ali**, Kenya Hospices and Palliative Care Association, Nairobi, Kenya; **Rumalie A. Corvera**, Asian Hospital and Medical Centre, Muntinlupa, Philippines; **Henry D. Ddungu**, Uganda Cancer Institute and Hutchinson Center Research Institute, Kampala, Uganda; **Liliana De Lima**, International Association for Hospice and Palliative Care, Houston, TX; **Maria Del Pilar Estevez-Diz**, Instituto do Câncer do Estado de São Paulo, São Paulo, Brazil; **Frank D. Ferris**, OhioHealth, Columbus, OH; **Nahla Gafer**, Radiation and Isotope Center, Khartoum, Sudan; **Harmala K. Gupta**, CanSupport, New Delhi, India; **Susan Horton**, University of Waterloo, Waterloo, Ontario, Canada; **Graciela Jacob**, Instituto Nacional de Cancerología, Buenos Aires, Argentina; **Ruinuo Jia**, Henan University of Science and Technology, Luoyang, People’s Republic of China; **Frank L. Lu**, National Taiwan University Children's Hospital, Taipei, Republic of China; **Daniela Mosoiu**, Transylvania University Brasov, Brasov, Romania; **Christina Puchalski**, George Washington University, Washington, DC; **Carole Seigel**, Massachusetts General Hospital Cancer Center, Boston, MA; **Olaitan Soyannwo**, University College Hospital Ibadan, Ibadan, Nigeria; and **James F. Cleary**, University of Wisconsin Carbone Cancer Center, Madison, WI.

## Abstract

**Purpose:**

The purpose of this new resource-stratified guideline is to provide expert guidance to clinicians and policymakers on implementing palliative care of patients with cancer and their caregivers in resource-constrained settings and is intended to complement the *Integration of Palliative Care Into Standard Oncology Care: American Society of Clinical Oncology Clinical Practice Guideline Update* of 2016.

**Methods:**

ASCO convened a multidisciplinary, multinational panel of experts in medical oncology, family medicine, radiation oncology, hematology/oncology, palliative and/or hospice care, pain and/or symptom management, patient advocacy, public health, and health economics. Guideline development involved a systematic literature review, a modified ADAPTE process, and a formal consensus-based process with the Expert Panel and additional experts (consensus ratings group).

**Results:**

The systematic review included 48 full-text publications regarding palliative care in resource-constrained settings, along with cost-effectiveness analyses; the evidence for many clinical questions was limited. These provided indirect evidence to inform the formal consensus process, which resulted in agreement of ≥ 75% (by consensus ratings group including Expert Panel).

**Recommendations:**

The recommendations help define the models of care, staffing requirements, and roles and training needs of team members in a variety of resource settings for palliative care. Recommendations also outline the standards for provision of psychosocial support, spiritual care, and opioid analgesics, which can be particularly challenging and often overlooked in resource-constrained settings. Additional information is available at www.asco.org/resource-stratified-guidelines.

It is the view of ASCO that health care providers and health care system decision makers should be guided by the recommendations for the highest stratum of resources available. The guideline is intended to complement but not replace local guidelines.

## INTRODUCTION

American Society of Clinical Oncology (ASCO) is committed to the integration of palliative care in oncology^1-5^ and recognizes differences in access to services, especially specialist palliative care, across settings. The purpose of this new resource-stratified guideline is to provide expert guidance to clinicians and policymakers on implementing palliative care in resource-constrained settings and is intended to complement the *Integration of Palliative Care Into Standard Oncology Care: American Society of Clinical Oncology Clinical Practice Guideline Update* of 2016. Most of the research on which the nonresource-constrained guidelines were based was conducted in maximal resource institutions, for example, in the United States, Canada, and Britain.

Research on palliative care began relatively recently in all settings, but much work is still needed to ensure that palliative care in low- and middle-income countries (LMICs) and other resource-constrained settings is based on a rigorous and relevant research base.^6^ The 2016 Guideline Update reviewed the lack of palliative care information and research in underserved communities in the United States and that patients identified as white have been over-represented in palliative care research. Most evidence and guidelines (Table 1) come from high-income countries (HICs)^1^; research findings related to specialist-based interventions in tertiary care centers in HICs can often not be readily extrapolated to resource-constrained or resource-poor settings, and “there is a great need for evidence-based regional and local research that takes into account the location and nature of care, access to medical and nonmedical resources, community attitudes and support, distinct cultural milieus, and multiple other local factors.”^7^^(p66s)^

**Table 1:**
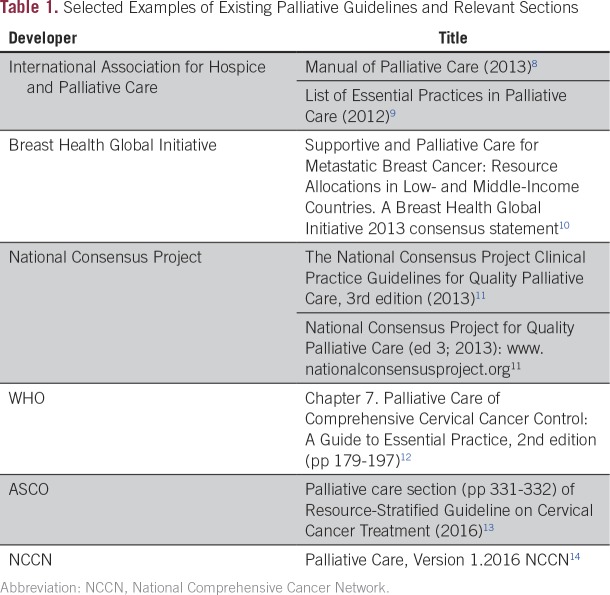
Selected Examples of Existing Palliative Guidelines and Relevant Sections

As this Expert Panel’s literature search confirms and others have observed, most research from LMICs is observational and descriptive. Reasons for this include the lack of resources, training, and interest to conduct research and the higher priority of providing palliative care itself. There are some moves to develop instruments that could be used in research, for example, the African Palliative Care Association developed the African Palliative Outcome Scale with collaborators from Kings College.^15^

Therefore, this ASCO guidance makes expert consensus recommendations regarding implementing aspects on, for example, the personnel, training, workforce, model, and timing of palliative care in resource-constrained settings (Table 2 describes the levels of settings used in this guideline). In addition, it complements the non–resource-constrained ASCO guideline by examining the role of specific personnel, including nurses, spiritual care providers, and counselors.

**Table 2:**
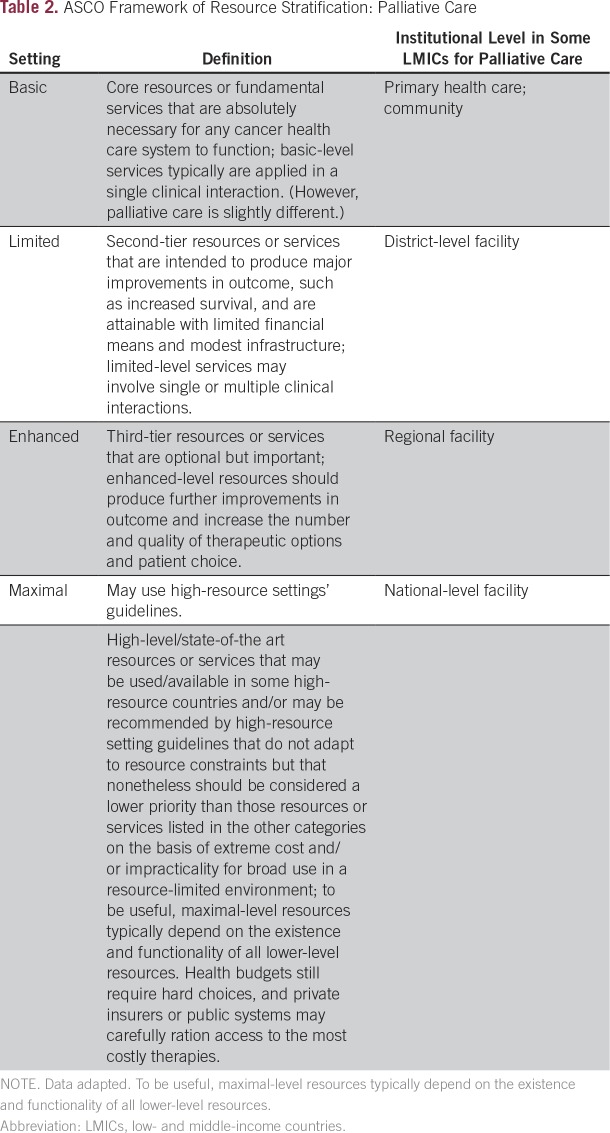
ASCO Framework of Resource Stratification: Palliative Care

“Multidisciplinary team approach" refers to a patient care model that includes experts from different disciplines, whereas an “interdisciplinary team approach” requires a more integrated and coordinated approach to patient care, where experts from different disciplines establish shared patient care goals for a more holistic approach to patient care. As in the *Integration of Palliative Care Into Standard Oncology Care* Guideline Update: “In this guideline, a family caregiver is defined as either a friend or a relative whom the patient describes as the primary caregiver; it may be someone who is not biologically related” and this guideline recognizes there are cultural variations in the definition of families/primary caregivers.^1^^(pp96,97)^

**THE BOTTOM LINE**

**Palliative Care in the Global Setting: American Society of Clinical Oncology Resource-Stratified Practice Guideline**

**Guideline Question**

In each setting, what are the most practical models of delivery of palliative care to patients with cancer and their caregivers?

**Target Population**

Patients with cancer, both adults and children, and their family caregivers

**Target Audience**

Clinicians, patients, caregivers, palliative care specialists, health planners, policymakers, hospices, health care institutions, community health workers, spiritual care providers

**Methods**

A multinational, multidisciplinary Expert Panel was convened to develop palliative care practice guideline recommendations based on a systematic review of medical literature and formal consensus.

Authors’ note: It is the view of ASCO that health care providers and health care system decision makers should be guided by the recommendations for the highest stratum of resources available. The guidelines are intended to complement, but not replace, local guidelines.

**Recommendations**

**1. Palliative Care Models**

**Recommendation 1.0 General:**

There should be a coordinated system where the palliative care needs of patients and families are identified and met at all levels, in collaboration with the team providing oncology care. The health care system should have trained personnel who are licensed to prescribe, deliver, and dispense opioids at all levels. Distance communication should be instituted at the national or regional level through oncology centers (or other tertiary care centers) to support those providing oncology care to patients in lower resource areas (Type of recommendation: formal consensus; not rated).

**Recommendation 1.1 Basic (Primary Health Care):**

Palliative care needs should be addressed in the community or at the primary health care center. These needs may be addressed by primary health care providers, nurses, community health workers, volunteers, and/or clinical officers (Type of recommendation: evidence based and formal consensus; Evidence quality: intermediate; Strength of recommendation: moderate).

**Recommendation 1.2 Limited (District):**

In addition to provision of palliative care in the community and at primary health care centers, outpatient palliative care services should be established. When a counselor is not available, psychosocial and spiritual needs may be addressed by team members trained in basic palliative care (Type of recommendation: formal consensus; Evidence quality: intermediate; Strength of recommendation: moderate).

**Recommendation 1.3 Enhanced (Regional):**

In addition to the community-based and outpatient palliative care services available at the limited level, inpatient consultation services should be available to hospitalized patients with palliative care needs. Consultation services should be provided by an interdisciplinary team, including (but not limited to) a physician, nurse, counselor, and pharmacist. Mental health and spiritual services may be added to the team when possible (Type of recommendation: formal consensus; Evidence quality: intermediate; Strength of recommendation: strong).

**Recommendation 1.4 Maximal (National):**

In addition to the palliative care services available at the enhanced level, dedicated inpatient palliative care beds should be established, staffed with trained professionals. No oncology center, hospice, or palliative care facility should exist without a well-developed palliative care team, with its different specialties (Type of recommendation: formal consensus; not rated).

**2. Timing**

**Recommendation 2.0 General:**

Palliative care needs should be addressed for all patients with cancer at presentation using appropriate screening, especially when disease-modifying interventions are not available (Type of recommendation: formal consensus; not rated).

**Recommendation 2.1 Basic and Limited:**

The palliative care needs of patients with cancer should be addressed early in the course of illness by existing health professionals trained in the basics of palliative care.

**Recommendation 2.1 Basic and Limited:**

The palliative care team should address the needs of all patients with cancer, at a minimum:

- Patients with overwhelming symptoms, whether physical, psychological, or spiritual
- Patients who develop metastasis, regardless of the type of cancer
- Patients who cannot receive active treatment with curative or life-prolonging intent
- Patients with malignancies with limited life expectancy, eg, hepatocellular carcinoma

**Recommendation 2.1 Basic and Limited:**

(Type of recommendation: formal consensus; Evidence quality: intermediate; Strength of recommendation: weak).

**Recommendation 2.2 Enhanced and Maximal:**

Please note, this recommendation is from the *Integration of Palliative Care Into Standard Oncology Care: American Society of Clinical Oncology Clinical Practice Guideline Update*, *Journal of Clinical Oncology*, 2016; the only change is in bold.^1^

**Enhanced and Maximal:**

For newly diagnosed patients with advanced cancer, the Expert Panel suggests a modification of the non–resource-stratified guideline recommendation to early palliative care **team** involvement, starting early in the diagnosis process and ideally within 8 weeks of diagnosis.

**Enhanced and Maximal:**

Note: In maximal resource settings, the intent is to provide concurrent antitumor therapy and referral to interdisciplinary palliative care teams (Type of recommendation: informal consensus; Evidence quality: intermediate; Strength of recommendation: moderate).

**3. Workforce, Knowledge, and Skills**

**Recommendation 3.1 Basic (Primary Health Care Provider):**

All health professionals should be trained in basic palliative care skills. This basic training should include identifying the palliative care needs of patients and their families, communication skills, assessment and management of pain and other symptoms, supportive care, and prescribing and/or dispensing of medications at a level appropriate to responsibilities. These needs may be addressed by primary health care providers, nurses, community health workers, volunteers, and/or clinical officers. If trained professionals are not available at the local level, health professionals should seek distance consultation and referral where appropriate (Type of recommendation: evidence based and formal consensus; Evidence quality: intermediate; Strength of recommendation: strong).

**Recommendation 3.2 Limited (District-Level Facility):**

Interdisciplinary teams are the core of palliative care. At this level, teams should include at least a nurse and a medical officer who have palliative care training. An appropriately trained counselor should be added, if available. Team members should be trained to assess, diagnose, and deliver care. All specialists in fields relevant to palliative care should have the knowledge and skills described in the basic level plus additional training in symptom assessment and management, communication issues, and psychosocial and spiritual needs. Oncologists practicing at this level should have basic training in palliative care. This level may or may not have professionals with formal advanced training in palliative care (Type of recommendation: evidence based and formal consensus; Evidence quality: intermediate; Strength of recommendation: strong).

**Recommendation 3.3 Enhanced (Regional Facility):**

A team including a physician, a nurse, and a counselor should provide palliative care. The team should include a health care provider trained to prescribe and with access to someone licensed to dispense medications. In the absence of a palliative care specialist, oncologists at this level should be trained in basic palliative care. All health professionals in relevant fields should have the knowledge and skills described in Recommendations 1.1 and 1.2, plus enhanced skills on how to treat refractory symptoms and how to manage emotional crisis and existential distress. This level should include professionals with formal advanced training and education in palliative care, such as (but not limited to) physicians, nurses, counselors, and pharmacists (Type of recommendation: evidence based and formal consensus; Evidence quality: intermediate; Strength of recommendation: strong).

**4. Nurse Role in Pain Management**

**Recommendation 4.0 (Across All Settings):**

The nurse should participate in ensuring care coordination and meeting patient and family needs. Where permitted, appropriately trained nurses may prescribe medicines, including controlled medicines. Nurses should be trained to assess patients’ palliative care needs, including pain control assessment and evaluation (as well as other knowledge and skills described in recommendations under Palliative Care Models recommendations), make recommendations, and communicate needs to adequately trained health care providers who are permitted to prescribe (Type of recommendation: evidence based; Evidence quality: intermediate; Strength of recommendation: strong).

**5. Spiritual Care**

**Recommendation 5.0 (Across All Settings):**

Spiritual care provided by appropriately trained providers should be available in all settings, whether locally or by referral. In addition to providing direct patient care, spiritual care providers may advise and support the care team to support the patients and their families. Nurses or counselors may be trained to assess the spiritual needs of patients and their families. Providers should be observant of and sensitive to the religious norms of patients and families (Type of recommendation: formal consensus; Evidence quality: insufficient; Strength of recommendation: weak).

**6. Social Work/Counseling**

**Qualifying statement:**

The psychosocial needs of patients and their families should be addressed in all settings and across the cancer care continuum. This role can be addressed by social workers, mental health professionals, or community health workers with training in the needs of palliative care patients and the special approaches required in this population. The role of the social worker/counselor becomes more critical in patients with advanced illness or when curative therapies are not an option, as is often the case in limited-resource settings. Collaboration with counselors/social workers can assist with communication between patients and clinicians, when available.

**Recommendation 6.1 Basic:**

When staffing is limited and specialized counselors are not available, physicians and/or nurses may play this role. They should receive the training to provide psychosocial care and be given enough time with the patient to allow them to provide it (Type of recommendation: formal consensus; Evidence quality: insufficient; Strength of recommendation: weak).

**Recommendation 6.2 Limited:**

Physicians and nurses may address the psychosocial needs of patients and families and should receive the training to do so. If possible, a social worker/counselor/volunteer/spiritual care provider should be available to attend to patients and families with a high burden of psychosocial issues (Type of recommendation: formal consensus; Evidence quality: insufficient; Strength of recommendation: weak).

**Recommendation 6.3 Enhanced:**

Counselors with special training in palliative care should be core members of the palliative care interdisciplinary team to provide psychosocial services to patients and families (Type of recommendation: formal consensus; Evidence quality: insufficient; Strength of recommendation: weak).

**7. Opioid Availability**

***Recommendation 7.0* General *(Across All Settings):***

Health care systems should safely provide opioids and ensure that the supply is readily and continually available for dispensing by trained professionals and accessible to patients to meet their needs, following the principles of balance through regulations, policy, and existing recommendations. Health care systems should strive to offer all pain control interventions on the WHO Essential Medicines List (Type of recommendation: formal consensus; Evidence quality: intermediate; Strength of recommendation: moderate).

**Recommendation 7.1 Basic:**

Local health care institutions should have access to immediate release (IR) oral and injectable morphine to address the pain needs of patients with cancer as assessed, prescribed, and dispensed by appropriately trained health care providers (Type of recommendation: formal consensus; Evidence quality: intermediate; Strength of recommendation: moderate).

**Recommendation 7.2 Limited:**

In addition to IR oral and injectable morphine available at the basic level, sustained-release morphine should be available in limited-resource level settings. Health care systems at the limited-resource level should be able to prescribe and dispense these three forms of morphine (intravenous, IR, and sustained release; Type of recommendation: formal consensus; Evidence quality: intermediate; Strength of recommendation: moderate).

**Recommendation 7.3 Enhanced:**

In addition to the opioids available at the limited-resource level, fentanyl and methadone (WHO Essential Medicines List) should be available for pain management (Type of recommendation: formal consensus; Evidence quality: intermediate; Strength of recommendation: moderate).

**All recommendations underwent formal consensus.**

***Additional Resources***

More information, including a Data Supplement with additional evidence tables, a Methodology Supplement with information about evidence quality and strength of recommendations, slide sets, and clinical tools and resources, is available at www.asco.org/resource-stratified-guidelines. Patient information is available at www.cancer.net.

**ASCO believes that cancer clinical trials are vital to inform medical decisions and improve cancer care, and that all patients should have the opportunity to participate.**

## GUIDELINE QUESTIONS

This clinical practice guideline addresses seven overarching questions: (1) Who should provide palliative care in the absence of specialized palliative care physicians, and what is the minimum training necessary to meet the palliative care needs of the community (whether by an oncologist or other clinicians/providers)? (2) What is the most effective model of palliative care delivery? (3) When is the best time to involve a palliative care team in cancer care? (4) What is the role of the nurse in palliative care assessment, pain and symptom control, and drug prescriptions (opioid prescriptions)? (5) At what level of health care (health centers, dispensary, hospitals) should oral opioids be available? (6) What is the place of spiritual care in palliative care? (7) What are the roles of social workers/counselors in palliative care?

## METHODS

### Guideline Development Process

This systematic review-based guideline product was developed by a multidisciplinary Expert Panel, which included two patient representatives and ASCO guidelines staff with health research methodology expertise (Appendix Table A1). The Expert Panel met via teleconference and in person and corresponded through e-mail. Based upon the consideration of the evidence, the authors were asked to contribute to the development of the guideline, provide critical review, and finalize the guideline recommendations. Members of the Expert Panel were responsible for reviewing and approving the penultimate version of the guideline, which was then circulated for external review and submitted to a peer-reviewed journal for editorial review and consideration for publication. This guideline was partially informed by ASCO’s modified Delphi Formal Expert Consensus methodology, during which the Expert Panel was supplemented by additional experts recruited to rate their agreement with the drafted recommendations. The entire membership of experts is referred to as the Consensus Panel (the Data Supplement provides a list of members). All ASCO guidelines are ultimately reviewed and approved by the Expert Panel and the ASCO Clinical Practice Guideline Committee prior to publication. This guideline adaptation was also informed by the ADAPTE methodology and consensus processes used together as an alternative to de novo guideline development. Adaptation of guidelines is considered by ASCO in selected circumstances, when one or more quality guidelines from other organizations already exist on the same topic. The objective of the ADAPTE process is to take advantage of existing guidelines to enhance the efficient production, reduce duplication, and promote the local uptake of quality guideline recommendations. All funding for the administration of the project was provided by ASCO.

The recommendations were developed by using a literature review and Expert Panel members’ expertise. Articles were selected for inclusion in the systematic review of the evidence based on the following criteria:

- Population: patients with cancer
- Assess an intervention
- Phase II or III randomized trials or quasi-randomized, controlled before-after, or prospective cohort studies.

ASCO’s adaptation and formal consensus processes begin with a literature search to identify candidate guidelines for adaptation. Cochrane Systematic Review and National Guideline Clearinghouse databases were searched for guidelines, systematic reviews, and meta-analyses published between 1966 and 2016. The Panel used literature searches (1966-2017), existing guidelines and expert consensus publications, literature suggested by the Panel, and clinical experience as guides. Inclusion criteria identified publications that were (1) on palliative care interventions in resource-constrained settings, (2) guidelines developed by multidisciplinary content experts as part of a recognized organizational effort, and (3) published between 1966 and 2017. Searches for cost-effectiveness analyses were also conducted. Articles were excluded from the systematic review if they were (1) meeting abstracts, phase I trials, retrospective studies, and (2) books, editorials, commentaries, letters, news articles, case reports, or narrative reviews.

The guideline recommendations were crafted, in part, using the Guidelines Into Decision Support (GLIDES) methodology and accompanying BRIDGE-Wiz software.^16^ In some selected cases where evidence was lacking, but there was a high level of agreement among the Expert Panel, informal consensus was used (as noted with the Recommendations). Detailed information about the methods used to develop this guideline is available in the Methodology Supplement and Data Supplement at www.asco.org/resource-stratified-guidelines.

The ASCO Panel and guidelines staff will work with co-chairs to keep abreast of any substantive updates to the guideline. On the basis of formal review of the emerging literature, ASCO will determine the need to update.

This is the most recent information as of the publication date. For updates, the most recent information, and to submit new evidence, please visit www.asco.org/resource-stratified-guidelines.

### Guideline Disclaimer

The Clinical Practice Guidelines and other guidance published herein are provided by ASCO to assist providers in clinical decision making. The information herein should not be relied upon as being complete or accurate, nor should it be considered as inclusive of all proper treatments or methods of care or as a statement of the standard of care. With the rapid development of scientific knowledge, new evidence may emerge between the time information is developed and when it is published or read. The information is not continually updated and may not reflect the most recent evidence. The information addresses only the topics specifically identified therein and is not applicable to other interventions, diseases, or stages of diseases. This information does not mandate any particular course of medical care. Further, the information is not intended to substitute for the independent professional judgment of the treating provider, as the information does not account for individual variation among patients. Recommendations reflect high, moderate, or low confidence that the recommendation reflects the net effect of a given course of action. The use of words like “must,” “must not,” “should,” and “should not” indicates that a course of action is recommended or not recommended for either most or many patients, but there is latitude for the treating physician to select other courses of action in individual cases. In all cases, the selected course of action should be considered by the treating provider in the context of treating the individual patient. Use of the information is voluntary. ASCO provides this information on an “as is” basis and makes no warranty, express or implied, regarding the information. ASCO specifically disclaims any warranties of merchantability or fitness for a particular use or purpose. ASCO assumes no responsibility for any injury or damage to persons or property arising out of or related to any use of this information, or for any errors or omissions.

### Guideline and Conflicts of Interest

The Expert Panel was assembled in accordance with ASCO’s Conflict of Interest Policy Implementation for Clinical Practice Guidelines (“Policy,” found at http://www.asco.org/rwc). All members of the Expert Panel completed ASCO’s disclosure form, which requires disclosure of financial and other interests, including relationships with commercial entities that are reasonably likely to experience direct regulatory or commercial impact as a result of promulgation of the guideline. Categories for disclosure include employment; leadership; stock or other ownership; honoraria, consulting or advisory role; speaker's bureau; research funding; patents, royalties, other intellectual property; expert testimony; travel, accommodations, expenses; and other relationships. In accordance with the Policy, the majority of the members of the Expert Panel did not disclose any relationships constituting a conflict under the Policy.

## RESULTS

A total of 48 full-text articles regarding resource-constrained settings were reviewed per the methodology described above. After reviewing these articles and observing the paucity of evidence from resource-constrained settings, as noted by other authors as well,^6^ the Steering Committee of the Expert Panel decided to use formal consensus informed by selections from these articles, as well as other literature suggested by the Panel. There was no literature on studies conducted in resource-constrained settings to inform practice for several of the clinical questions. Therefore, the panel chose to make consensus recommendations and relied on clinical experience, training, and judgment to formulate these recommendations, given that there were no conclusive data regarding this question. Studies cited are examples of literature on a given topic; however, the Panel does not intend the examples to convey the only or the highest levels of evidence.

During the first round of voting by the Consensus Panel, agreement with the individual recommendations ranged from 87.5% to 100% (N = 32 respondents). Although all the recommendations far exceeded the required 75% threshold, the guideline co-chairs chose to submit two recommendations to a second round of voting based on Consensus Panel comments. The revised recommendations underwent a second round of voting in which agreement with the recommendations ranged from 67% to 71% (N = 21 respondents). Results for each recommendation and each round of voting are provided in the Methodology Supplement.

## RECOMMENDATIONS

### CLINICAL QUESTION 1 (PALLIATIVE CARE MODELS)

What is the most effective model of palliative care delivery?

#### Recommendation 1.0 General:

There should be a coordinated system where the palliative care needs of patients and families are identified and met at all levels, in collaboration with the team providing oncology care. The health care system should have trained personnel who are licensed to prescribe, deliver, and dispense opioids at all levels. Distance communication should be instituted at the national or regional level through oncology centers (or other tertiary care centers) to support those providing oncology care to patients in lower resource areas (Type of recommendation: formal consensus; not rated).

#### Recommendation 1.1 Basic (Primary Health Care):

Palliative care needs should be addressed in the community or at the primary health care center. These needs may be addressed by primary health care providers, nurses, community health workers, volunteers, and/or clinical officers (Type of recommendation: evidence based and formal consensus; Evidence quality: intermediate; Strength of recommendation: moderate).

#### Recommendation 1.2 Limited (District):

In addition to provision of palliative care in the community and at primary health care centers, outpatient palliative care services should be established. When a counselor is not available, psychosocial and spiritual needs may be addressed by team members trained in basic palliative care (Type of recommendation: formal consensus; Evidence quality: intermediate; Strength of recommendation: moderate).

#### Recommendation 1.3 Enhanced (Regional):

In addition to the community-based and outpatient palliative care services available at the limited level, inpatient consultation services should be available to hospitalized patients with palliative care needs. Consultation services should be provided by an interdisciplinary team, including (but not limited to) a physician, nurse, counselor, and pharmacist. Mental health and spiritual services may be added to the team when possible (Type of recommendation: formal consensus; Evidence quality: intermediate; Strength of recommendation: strong).

#### Recommendation 1.4 Maximal (National):

In addition to the palliative care services available at the enhanced level, dedicated inpatient palliative care beds should be established, staffed with trained professionals. No oncology center, hospice, or palliative care facility should exist without a well-developed palliative care team, with its different specialties (Type of recommendation: formal consensus; not rated).

##### Literature Review and Analysis/Discussion:

Downing et al^17^ evaluated seven palliative care programs in Kenya^3^ and Malawi,^4^ using mixed methods, including case studies, literature review, an audit tool, and observations. The article outlined models for Specialist, District, and community-level palliative care.

Downing et al^17^^(p365)^ concluded the benefit of involvement of “community volunteers or home-based care assistants” at a level similar to the recommendations the ASCO Expert Panel made for basic-setting palliative care, in addition to nurses leading palliative care in a limited setting with potential volunteer involvement.

In that model, the Health Center/Community Level included: (1) pain and symptom assessment and management, (2)holistic care provision, (3) counseling and support for patients and families, (4) bereavement support, (5) social and nutritional support, (6) use of local resources, and (7) medications, if available, at no cost to patients. The emphasis is on home-based care, including outpatient care, led by nurses, and including volunteers. (This agrees with the ASCO basic recommendation—primary health care workers, community health outreach workers, and/or clinical officer—outpatient or home.) There are other existing models for home-based, community-supported palliative care, notably in the state of Kerala, India. Palliative care in Kerala linked a system of outreach clinics, all with access to morphine with care involving family caregivers and volunteers. There have been several publications on the Kerala system, a WHO demonstration project, although not formal evaluations,^18-21^ as summarized by Hannon et al^8^ in *JCO*, 2016. Other support for including volunteers in community-based/supported palliative care comes from Kenya, Malawi, and Uganda that was shown to “improve access to services; control of physical, emotional, and spiritual symptoms; and community attitudes toward the dying.”^7^^(p64),^^22^

According to Downing et al,^17^ the District Hospital Level (concordant with this guideline’s limited level) should include what is in the other levels plus adherence support for treatment. It may be led by a nurse, clinical officer, or physician depending on availability. There may also be support staff and volunteers (home care). (*Note:* This agrees with the ASCO limited recommendation for outpatient-based care.)

Also, in Downing et al,^17^ the Specialist Level included additional components to a physician-led hospital-based palliative care team and included rounds and team meetings; nurses did a first-level review, working with other teams; there was not an on-call service. Nurses may be clinical officers. (Note: Agrees with ASCO Enhanced—consult, inpatient, outpatient [latter optional]—adds inpatient here and Maximal—ASCO says dedicated inpatient palliative care beds/specialized team. This also agrees with the ASCO non–resource-stratified Maximal recommendation—all four levels should exist: home, outpatient, inpatient consult, inpatient unit.)^1^

The literature review found several descriptive studies with examples of community participation in community-based palliative care, including in India. See Table 1 in the Data Supplement for a list.

One example of a study the Panel discussed is a descriptive study by Devi et al^23^ of using home-based care in Sarawak, Malaysia, to extend a strong primary health care network; similar systems would likely need preexisting primary health care networks. The palliative care providers included at least a physician and a nurse trained in basic palliative care (and counselor and pharmacists, if available). The program included training, nurse empowerment, simplified referrals, access to medications, and pain reduction interventions. Facilitators included political support and public awareness. While the program trained > 1,200 clinicians, most were nonphysicians (and physicians had high turnover). The results were descriptive and included numbers of those trained and those receiving care. There was a low refusal rate by care recipients.

##### Discussion:

Different models of palliative care delivery include home care, outpatient, inpatient consult, and inpatient unit models. Although most of the studies found in the literature are descriptive, they report positive outcomes. ASCO recommends community-based and home care in Basic; outpatient- (the development of), home-, and hospital-based consult in Limited; home-based, consultation, and inpatient beds in Enhanced; and all four in Maximal. The Panel believes that health care institutions should have care/case manager(s) in place when and where it is feasible.

Some models can use components of other models, such as referrals, making the patient journey cyclical/multidirectional and remaining flexible and dynamic, while making the model more patient-centered at the community level (*v* provider centered) and developing more role definition and training for community-level health care providers.^17^ It is important to consider the policy environment, public support and awareness, resources, access to essential medicines, and training of health professionals.

### CLINICAL QUESTION 2 (TIMING)

When is the best time to involve a palliative care team in cancer care?

#### Recommendation 2.0 General:

Palliative care needs should be addressed for all patients with cancer at presentation using appropriate screening, especially when disease-modifying interventions are not available (Type of recommendation: formal consensus; not rated).

#### Recommendation 2.1 Basic and Limited:

The palliative care needs of patients with cancer should be addressed early in the course of illness by existing health professionals trained in the basics of palliative care.

The palliative care team should address the needs of all patients with cancer, at a minimum those with:

- Patients with overwhelming symptoms, whether physical, psychological, or spiritual
- Patients who develop metastasis, regardless of the type of cancer
- Patients who cannot receive active treatment with curative or life-prolonging intent
- Patients with malignancies with limited life expectancy, eg, hepatocellular carcinoma

(Type of recommendation: formal consensus; Evidence quality: intermediate; Strength of recommendation: weak).

#### Recommendation 2.2 Enhanced and Maximal:

Please note, this recommendation is from the *Integration of Palliative Care Into Standard Oncology Care: American Society of Clinical Oncology Clinical Practice Guideline Update*, JCO, 2016; the only change is in bold.^1^

##### Enhanced and Maximal:

For newly diagnosed patients with advanced cancer, the Expert Panel suggests a modification of the non–resource-stratified guideline recommendation to early palliative care **team** involvement, starting early in the diagnosis process and ideally within 8 weeks of diagnosis.

Note: In maximal resource settings, the intent is to provide concurrent antitumor therapy and referral to interdisciplinary palliative care teams (Type of recommendation: informal consensus; Evidence quality: intermediate; Strength of recommendation: moderate).

##### Literature Review and Analysis/Discussion:

This recommendation is supported by the ASCO non–resource-stratified guideline and other documents, eg, End-of-Life Care Policy: An Integrated Care Plan for the Dying, from the Indian Society of Critical Care Medicine and the Indian Association of Palliative Care.^24^ We did not identify any additional studies related to timing of referral in resource-constrained settings and therefore depend on the literature used in the ASCO non–resource-stratified palliative care guideline.^1^ The non–resource-stratified guideline included the recommendation that “For newly diagnosed patients with advanced cancer, the Expert Panel suggests palliative care service involvement, starting early in the diagnosis process and ideally within 8 weeks of diagnosis (Type: informal consensus; Evidence quality: intermediate; Strength of recommendation: moderate).” The Resource-Stratified Guideline palliative care Expert Panel emphasizes the addition of the subject word “palliative care team” to highlight the role of the team. Examples of screening tools are listed in the Maximal Resource Settings and given in Data Supplement (National Comprehensive Cancer Network Distress Thermometer, Edmonton Symptom Assessment Scale, Condensed Memorial Symptom Assessment Scale, Brief Pain Inventory).

Evidence is emerging, for some diseases, that specialist palliative care teams may also benefit those diagnosed with early-stage disease, in addition to those with advanced disease. The panel stresses the additional comment that 8 weeks is the maximum time within which clinicians should make referrals to palliative care services, but earlier referral is preferable.

### CLINICAL QUESTION 3 (WORKFORCE, KNOWLEDGE, AND SKILLS):

Who should provide palliative care in the absence of specialized palliative care physicians? What is the minimum training necessary to meet the palliative care needs of the community (whether by an oncologist or other clinicians/providers)?

#### Introduction:

This clinical question recognizes that outside of maximal settings, palliative care specialists are often not available. Some resource-constrained settings do not have oncologists, although this is changing in some settings. In Basic and Limited settings, palliative care is especially important, as patients often present at late stages and have greater palliative care needs.

#### Recommendation 3.1 Basic (Primary Health Care Provider):

All health professionals should be trained in basic palliative care skills. This basic training should include identifying the palliative care needs of patients and their families, communication skills, assessment and management of pain and other symptoms, supportive care, and prescribing and/or dispensing of medications at a level appropriate to responsibilities. These needs may be addressed by primary health care providers, nurses, community health workers, volunteers, and/or clinical officers. If trained professionals are not available at the local level, health professionals should seek distance consultation and referral where appropriate (Type of recommendation: evidence based and formal consensus; Evidence quality: intermediate; Strength of recommendation: strong).

#### Recommendation 3.2 Limited (District-Level Facility):

Interdisciplinary teams are the core of palliative care. At this level, teams should include at least a nurse and a medical officer who have palliative care training. An appropriately trained counselor should be added if available. Team members should be trained to assess, diagnose, and deliver care. All specialists in fields relevant to palliative care should have the knowledge and skills described in the basic level plus additional training in symptom assessment and management, communication issues, and psychosocial and spiritual needs. Oncologists practicing at this level should have basic training in palliative care. This level may or may not have professionals with formal advanced training in palliative care (Type of recommendation: evidence based and formal consensus; Evidence quality: intermediate; Strength of recommendation: strong).

#### Recommendation 3.3 Enhanced (Regional Facility):

A team including a physician, a nurse, and a counselor should provide palliative care. The team should include a health care provider trained to prescribe and access to someone licensed to dispense medications. In the absence of a palliative care specialist, oncologists at this level should be trained in basic palliative care. All health professionals in relevant fields should have the knowledge and skills described in Recommendations 1.1 and 1.2, plus enhanced skills on how to treat refractory symptoms and how to manage emotional crisis and existential distress. This level should include professionals with formal advanced training and education in palliative care, such as (but not limited to) physicians, nurses, counselors, and pharmacists (Type of recommendation: evidence based and formal consensus; Evidence quality: intermediate; Strength of recommendation: strong).

#### Recommendation 3.4 Maximal (National Cancer Center/Institute)—see the Ferrell et al^1^ guideline.

##### Literature Review and Analysis/Discussion:

There is evidence from several studies conducted in resource-constrained settings that health care personnel other than specialized palliative care physicians can provide basic palliative care when the latter are not available.

In settings with a limited number of adequately trained physicians, palliative care can be successfully provided by nurses or even laypeople (ie, nonmedically trained persons) given the right support and training. In many palliative care studies, interventions by nonmedically trained caregivers and nurse-led interventions have been found to have a positive impact. These include several cited in the ASCO non–resource-stratified guideline.^1^ This is confirmed in the literature found for the current guideline, which included primarily observational studies. (The role of nurses will be discussed in more detail in Clinical Question 4.)

Literature on family caregivers supports the concept that lay (also known as nonmedically trained) people (such as volunteers or community health workers) can be trained to provide care and have a positive impact on the patients. Some studies evaluating the impact of family caregivers participating in the provision of palliative care were included in the non–resource-stratified ASCO guideline. Family caregivers often provide hands-on primary care to patients in the community setting. One of the only randomized controlled trials (RCTs) found in the literature search for this resource-stratified guideline was a small RCT conducted in a non-US HIC, Singapore.^25^ Family caregivers are impacted by the illness experience, and palliative care aims to address the psychosocial needs not only of patients, but also of family members. This small, randomized, controlled trial included four home hospice organizations and an outpatient clinic in Singapore. Eighty caregivers were randomly assigned to experimental and standard care groups, and received a psychoeducational intervention to enhance their quality of life (QoL); the outcomes showed statistically significant improvements in QoL measures. The authors attributed the effective results to increases in understanding of four phases of anticipatory grief and the importance of caregiver-patient communication (according to this study, not well studied). Limitations of this study included the convenience sample, language barriers (English only), selection bias, and a small sample size.

In another example, Kristanti et al^26^ had nurse educator(s) train family caregivers in basic patient care, such as oral care, in an Indonesian pilot study. In this prospective quantitative study, there was an increase in the primary measure, patient QoL.

Nongovernmental/voluntary organizations (NGOs) are also involved in providing palliative care, especially at the community level. Evidence on NGO-provided palliative care in resource-constrained settings is limited. The literature search found a multimethod review that included narrative summaries of literature and qualitative research, such as key informant interviews^27^; however, it did not find any formal evaluations.

Several other guidelines and authors have suggested particular subjects for education and training for health care personnel in palliative care. Examples^7,28,29^ include providing education in assessing and managing patients’ pain and other symptoms, recognition of metastases, communication skills, leadership skills, cultural communication,^28^ and basic oncology skills (Table 3 for training resources from Maximal settings).^29^

**Table 3:**
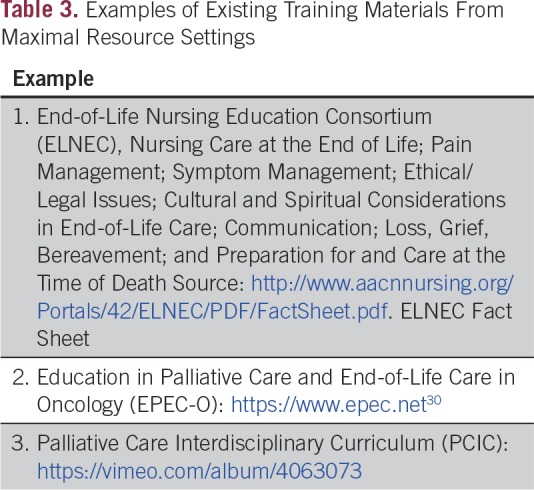
Examples of Existing Training Materials From Maximal Resource Settings

Integrating palliative care volunteers and community health workers and empowering the health care workforce, especially nurses (Clinical Question 4), is essential to achieve universal access to palliative care services in settings where access to physicians may be limited.

### CLINICAL QUESTION 4 (NURSE ROLE IN PAIN MANAGEMENT)

What is the role of the nurse in palliative care assessment, pain and symptom control, and medicine prescriptions, including controlled medicines (opioid prescriptions)?

#### Recommendation 4 (Across All Settings):

The nurse should participate in ensuring care coordination and meeting patient and family needs. Where permitted, appropriately trained nurses may prescribe medicines, including controlled medicines. Nurses should be trained to assess patients’ palliative care needs, including pain control assessment and evaluation (as well as other knowledge and skills described in recommendations under Clinical Question 1), make recommendations, and communicate needs to adequately trained health care providers who are permitted to prescribe (Type of recommendation: evidence based; Evidence quality: intermediate; Strength of recommendation: strong).

##### Literature Review and Analysis/Discussion:

Machira et al^31^ published a quasi-experimental program evaluation of registered nurse education on pain management in Kenya that included 31 nurses caring for adults with life-limiting illness in one hospital. “The study highlighted the fact that palliative care, specifically pain management, in Kenya has no critical mass that would provide mentorship and improve clinical practice. This suggests the need for formal palliative care and specifically pain management training for nurses at preregistration and as part of continuous professional development within clinical practice.”^31^^(p345)^ Nurses were either the focus of an intervention or on a team in many of the other studies reviewed, as well.

##### Nurse-Led Programs:

The role of nurses is paramount. Nurses should be able to carry out a basic palliative care assessment of the patient, follow-up, and communication. For complicated communication scenarios and symptom management, the nurse can assist the trained physician/psychologist/counselor/spiritual care providers. If nurses receive special training, they can be licensed to prescribe opioids. For example, a link-nurse program at Mulago Hospital (described in the next paragraph) increased nurses’ confidence in their ability to write morphine prescriptions in Uganda (although their role was limited to making recommendations to physicians, the latter of whom had prescribing ability).^32^

Downing et al^32^ conducted a mixed method study at Mulago Hospital Uganda, a tertiary hospital with hospital-based palliative care. They provided “generalist” palliative care training to 27 nurses, and outcomes included nurses’ confidence in providing palliative care. The study found changes in attitudes, developing new skills and knowledge, developing relationships, and an increase in access to and strengthening of palliative care. Challenges were found regarding writing morphine prescriptions, lack of interest by colleagues, and difficultly in convincing physicians of the value of palliative care.

A pilot qualitative study on a nurse-practitioner–led, general-practitioner–supported palliative care program was carried out in rural Australia. The study evaluated a single multidisciplinary case conference that found the nurse practitioners assumed prescribing of new medications or made dosing changes; there was a decrease in hospital utilization and an increase in the development of new advanced care plans as a result of this program.^33^

##### Discussion:

Along with the role of the oncologist trained in palliative care, an advanced practice nurse (APN) could play an important role in building and maintaining an interdisciplinary network of care, necessary for the management of complex palliative situations. The APN is a vital member of the interdisciplinary team and a key player who collaboratively integrates palliative practices throughout the patient's disease course by promoting QOL and reducing fragmented delivery of care. In the absence of trained palliative care physicians and oncologists, APNs could spearhead the development, implementation, and evaluation of palliative care services.

In resource-constrained settings, nurses are professionally the most important health workers who could excel in palliative care delivery system(s). Although the designation and licensing of APNs may not be an option in many countries, studies have shown that nurses can provide quality palliative care if they have the training to do so and the system is structured to allow them to play that role. Their role should not be limited to only the hospital, but also to the community and home care level and could be the linkage among patients, caregivers, physicians, and other interdisciplinary teams.

### CLINICAL QUESTION 5 (SPIRITUAL CARE)

What is the place of spiritual care in palliative care?

#### Recommendation 5.0 (Across All Settings):

Spiritual care provided by appropriately trained providers should be available in all settings, whether locally or by referral. In addition to providing direct patient care, spiritual care providers may advise and support the care team to support the patients and their families. Nurses or counselors may be trained to assess the spiritual needs of patients and their families. Providers should be observant of and sensitive to the religious norms of patients and families (Type of recommendation: formal consensus; Evidence quality: insufficient; Strength of recommendation: weak).

##### Discussion:

This section is based on expert opinion and formal consensus. Literature was not found by the systematic review from resource-constrained settings. Medical care providers should make appropriate referrals to certified chaplains or other spiritual support professionals. Professionals and volunteers interacting with patients/caregivers should use culturally appropriate language. This is critical for global work, as spiritual care professional/training needs to be developed from indigenous culture. Models such as from a US National Consensus Conference may need to be adapted or developed de novo in different cultures. As with other components of the medical history, a spiritual history is important for clinicians to take, especially during the initial consultation. If the patient describes difficulty with coping and/or that spiritual or religious resources are not working well for him or her, referral to a trained provider is advised.

In the Basic Model, spiritual care should be available, at the minimum, during end-of-life care and at bereavement. In the Limited Model, spiritual care should be available whenever patients and/or caregivers are in need for this aspect of necessary psychosocial support. In the Enhanced Model, whenever possible, spiritual care provided by appropriately trained providers may be involved as a part of the interdisciplinary team in cancer care as soon as diagnosis, including in helping to break bad news. This may be especially helpful when clinicians are initially communicating a diagnosis to patients and caregivers. Early involvement may help patients and families to cope with grief and receive help to reach optimal decisions.

Clinicians should document a patient’s spiritual/existential distress. All patients may be offered basic spiritual support, for example, giving a framework so they may consider goals and receive hope along with medical outcomes. Ongoing assessment and evaluation are suggested.

Psychosocial, spiritual, and bereavement support are key elements of palliative care. Most programs in Maximal settings use an interdisciplinary team that may include social workers, chaplains, psychiatrists, psychologists, and/or bereavement counselors. For many patients, spirituality plays an important role in coping with serious or terminal illnesses; spiritual distress is highly correlated with a desire for a hastened death.^34^ A growing body of literature (primarily from Maximal resource settings) supports the notion that spiritual care is a patient need. The data also suggest that patients' spiritual, religious, and cultural beliefs affect health care decision making and health care outcomes, including coping, QOL, and pain management. Studies have reported that spirituality and/or religion may be important to patients with cancer and may influence medical decision making.^35^ Research shows spirituality or religion impact QOL, coping, depression, and anxiety, and play a role in improved social functioning and maintaining social relationships.^36-39^ Spiritual care is a core domain of palliative care (eg, Domain 3: Spiritual and Cultural Assessment and Management of the ASCO/American Academy of Hospice and Palliative Medicine (AAHPM) statement).^2^ Spiritual care was one of four modules in the Ferrell et al^40^ and Sun^41^ papers described in the non–resource-stratified guideline,^1^ which also addressed Dignity Therapy, supported by various non–resource-constrained setting studies.^42,43^

However, while available data suggest both the central importance of spiritual concerns to seriously ill patients and families, it also indicates widespread failure of health care professionals to address this domain, perhaps because of lack of training or spiritual care capacity in many clinical settings. Spiritual assessment should be part of other assessments conducted in palliative care. There are recommended Standards for Spiritual Care.^44,45^ There are some examples from South Africa, Korea, Chile, and Mexico (C. Puchalski, personal communication, June 21, 2017).

Other guidelines from Maximal settings have commented on the role of spiritual care (Table 3 in Data Supplement 6).

### CLINICAL QUESTION 6 (SOCIAL WORK/COUNSELING)

What are the roles of social workers/counselors in palliative care?

#### Qualifying Statement:

The psychosocial needs of patients and their families should be addressed in all settings and across the cancer care continuum. This role can be addressed by social workers, mental health professionals, or community health workers with training in the needs of palliative care patients and the special approaches required in this population. The role of the social worker/counselor becomes more critical in patients with advanced illness or when curative therapies are not an option, as is often the case in limited-resource settings. Collaboration with counselors/social workers can assist communication between patients and clinicians, when available.

#### Recommendation 6.1 Basic:

When staffing is limited and specialized counselors are not available, physicians and/or nurses may play this role. They should receive the training to provide psychosocial care and be given enough time with the patient to allow them to provide it (Type of recommendation: formal consensus; Evidence quality: insufficient; Strength of recommendation: weak).

#### Recommendation 6.2 Limited:

Physicians and nurses may address the psychosocial needs of patients and families and should receive the training to do so. If possible, a social worker/counselor/volunteer/spiritual care provider should be available to attend to patients and families with a high burden of psychosocial issues (Type of recommendation: formal consensus; Evidence quality: insufficient; Strength of recommendation: weak).

#### Recommendation 6.3 Enhanced:

Counselors with special training in palliative care should be core members of the palliative care interdisciplinary team to provide psychosocial services to patients and families (Type of recommendation: formal consensus; Evidence quality: insufficient; Strength of recommendation: weak).

##### Discussion:

This section is based on clinical experience and formal consensus. No studies were found by the literature search from resource-constrained settings specifically on the intervention of the inclusion of social workers/dedicated counselors. However, counseling is a recognized domain of palliative care. Elements such as counseling, emotional support and assessment, care coordination, and patient education are woven throughout (part of the AAHPM-ASCO domains: communication and shared decision making, advance care planning, carer support, coordination and continuity of care, psychosocial assessment and management, spiritual and cultural assessment and management).^2^

Much of the discussion under Clinical Question 6 is relevant to the role of mental health professionals. Several studies reviewed to inform the Expert Panel discussion included counselors/mental health services. Please see Table 2 in Data Supplement 5 for relevant points from these studies.

### CLINICAL QUESTION 7 (OPIOID AVAILABILITY)

At what level of health care (health centers, dispensary, hospitals) should opioids (primarily oral) be available?

#### Introduction:

The regular administration of oral morphine to patients with advanced cancer improves QOL and relief of suffering. Morphine was included in the first WHO Essential Medicines List and Cancer Pain Relief, together with the WHO three-step ladder.^46^ Many barriers to patient access have been identified,^47^ and have been addressed by the WHO in guidelines on the principles of balance.^48,49^ While the consumption of medical opioids increased in higher income countries, there continue to be great disparities in medical opioid consumption when compared with low- and middle-income countries. This disparity has been highlighted since the 1990s in multiple publications, but recognized most recently by the World Health Assembly in its palliative care resolution of 2014^50^ and the United Nations General Assembly 2016,^51^ the International Narcotics Control Board,^52,53^ Disease Control Priorities 3 (http://dcp-3.org),^54^ and a Lancet Commission report of 2017.^55^ These references were identified outside of the systematic literature review.

#### Recommendation 7.0 General (Across All Settings):

Health care systems should safely provide opioids and ensure that the supply is readily and continually available for dispensing by trained professionals and accessible to patients to meet their needs, following the principles of balance through regulations, policy, and existing recommendations. Health care systems should strive to offer all pain control interventions on the WHO Essential Medicines List (Type of recommendation: formal consensus; Evidence quality: intermediate; Strength of recommendation: moderate).

#### Recommendation 7.1 Basic:

Local health care institutions should have access to immediate release (IR) oral and injectable morphine to address the pain needs of patients with cancer as assessed, prescribed, and dispensed by appropriately trained health care providers (Type of recommendation: formal consensus; Evidence quality: intermediate; Strength of recommendation: moderate).

#### Recommendation 7.2 Limited:

In addition to IR oral and injectable morphine available at the basic level, sustained-release morphine should be available in limited-resource level settings. Health care systems at the limited-resource level should be able to prescribe and dispense these three forms of morphine (intravenous, IR, and sustained-release); Type of recommendation: formal consensus; Evidence quality: intermediate; Strength of recommendation: moderate).

#### Recommendation 7.3 Enhanced:

In addition to the opioids available at the limited resource level, fentanyl and methadone (WHO Essential Medicines List) should be available for pain management (Type of recommendation: formal consensus; Evidence quality: intermediate; Strength of recommendation: moderate).

##### Literature Review and Analysis/Discussion:

While overall, resource-constrained settings have shown little increase in opioid consumption, there are examples that illustrate the impact that can be made in opioid availability in some countries.

One of the few articles potentially relevant to resource-constrained settings for this clinical question found in the literature search included patients with cancer hospitalized with pain in a palliative care and hospice ward of a Hong Kong public hospital. In this randomized, prospective study, patients participated in a pain management program provided by nurses. While pain scores showed no difference between groups, the use of as-needed-only analgesics was higher in the intervention group, as was the use of nonpharmacologic methods.^56^

The scope of this guideline does not include reviewing the literature on opioids for cancer pain relief. However, we will cite one systematic review from Cochrane,^57^ which was an update of previous Cochrane reviews conducted by Wiffen et al.^58-60^ It included seven new studies, but they did not meet criteria for updating their past systematic review, which had a total of 62 RCTs included, primarily investigating efficacy, specifically pain and pain relief and adverse events reported by patients with cancer. The authors assessed the evidence as being of poor quality and having a high risk of bias (in most studies); however, studies found that > 90% of participants experienced decreases in pain to “no worse than mild pain” and providers can “titrate with oral morphine of any formulation,” although they did not reveal which formulation was best.^57^

There is a large disparity between opioid availability in HICs and LMICs.^52,61^ The WHO Essential Medicines List now includes fentanyl skin patches and methadone, which provides support of the recommendation in this guideline for Enhanced settings.^62^

The WHO Palliative Care Strategy states that policymakers should address “medicine availability, education, and government policy”; WHO also provides a model on access to opioids. According to LeBaron et al,^29^ hospitals should have a first priority of making oral IR morphine available. “Institutions that have been successful in procuring morphine…could serve as role models and mentors to other institutions who wish to improve access to pain relief for their patients. Resources exist through international organizations, such as the Pain and Policy Studies Group, as well as regional institutions, such as the Indian Association of Palliative Care and Pallium India, to support improving access to opioids.”^29^^(p10)^

Other existing policies/recommendations advise finding balance between controlling opioids to prevent abuse and/or diversion and ensuring patients with cancer in pain have access. The WHO recommends that national policies establish a system “that prevents diversion *and* ensures adequate availability for medical use”^63,64^ and that the former should not impede the latter. The WHO Guidance is based on the principle of “balance,” which asserts that governments’ obligation to control narcotic drugs is not only to prevent drug abuse, but also to ensure the availability of opioid analgesics for medical purposes. Controls aimed at preventing drug abuse and diversion must not interfere with the adequate availability of opioid analgesics for patients’ pain relief.^49^

The International Drug Control Board states the importance of countries’ cooperation with WHO, United Nations Office on Drugs and Crime, and International Drug Control Board to insure this balance in international drug control conventions.^53^^(p4)^

## COST IMPLICATIONS

There are very few articles on the economics of palliative care in LMICs, and clearly the form of such care is different than in HICs. HICs have until recently provided such care primarily in institutions (hospitals, long-term care homes/nursing homes, and in growing popularity, hospices). Typically, care in the last year/last 3 months/last month can be quite costly to the health sector. There has been a move more recently in HICs to shift dying from hospitals to hospices or home (where most patients prefer to die). This can reduce costs to health care institutions/systems.

We can, however, extrapolate from the experience of HICs. Drug costs tend to be only a small fraction of care at the end of life (7% in Poland and 4.7% in France),^65^ with salaries tending to be the large majority of costs. Since salaries go up with national income, this gives an indication of the costs of care also in low-income countries. Expressing costs of care at a ratio to gross national income (GNI) per capita, cost in the last year of life is 170% of GNI per capita in Ireland,^66^ in the last year of life is 25% of GNI per capita in Netherlands (unless the individual also has dementia, in which case, it goes up to 100% of GNI per capita),^67^ and in the last 15 days of life in Poland, hospital care is 12% of GNI per capita^65^. In Thailand, the only LMIC for which data could be located, the societal cost was 71% of GNI per capita for the last 1 to 3 months of life for those with end-stage renal disease who were not on dialysis.^68^ There are many estimates for the United States, but these are likely not a good guide for other countries.

One difference between the HICs and Thailand (an upper-middle-income country) is that in the former, up to 75% of the total cost is paid by the public sector (eg, Brick et al^66^ provide estimates for Ireland), whereas in Thailand, only 25% of the cost falls on the government.^68^ Informal caregiving and out-of-pocket expenditures can be considerable and form a large share of the costs in LMICs.

Studies suggest that good planning and specialist expertise can both improve pain control for patients and allow them a greater possibility of dying at home (typically the preferred choice). This can be assisted by advance directives (provided that these are determined early enough and not as the patient arrives for the last time at the hospital) and where nurses coordinate palliative care.^69^

Summarizing the findings of these studies above suggests that for the most resource-constrained countries, the limiting factor for palliative care is not cost of drugs, but rather, the availability of trained nurses to coordinate care and support for family members who are likely to provide the majority of care. Having appropriately trained human resources available can cost a substantial fraction of national per capita income, per patient needing such care.

## LIMITATIONS OF THE RESEARCH AND FUTURE DIRECTIONS

There were limitations on the evidence to inform some of the recommendations, due to many recognizable factors, such as prioritization of patient care and limited funding and infrastructure for research. Limitations include:

- Results from resource-limited countries/regions
- There are few RCTs, and existing studies have low sample sizes
- Most of the studies have interventions, such as education packages on palliative care, for a week or less
- Lack of formal evaluation from places with experience (eg, Uganda)
- The quality of evidence regarding oral morphine is poor, according to the Cochrane Systematic Review

Future research should include the following principles:

- Nurses are the most preferred alternatives in the absence of physicians
- Along with caregivers and family, patient education and empowering pain management and self-care has beneficial effects
- In the absence or lack of registered nurses, programs like those with nursing aides can be effective with patients at the end of life
- Compulsory palliative care education in medical and nursing curricula with priority may be a long-term solution

## EXTERNAL REVIEW

The draft recommendations were released to the public for open comment from November 7 to November 22, 2017. A total of two respondents, who had not previously reviewed the recommendations, either agreed or agreed with slight modifications to the recommendations. Comments received were reviewed by the Expert Panel and integrated into the final draft before approval by the ASCO Clinical Practice Guideline Committee.

## GUIDELINE IMPLEMENTATION

ASCO guidelines are developed for implementation across health settings. Barriers to implementation include the need to increase awareness of the guideline recommendations among front-line practitioners and survivors of cancer and caregivers, and also to provide adequate services in the face of limited resources. The guideline Bottom Line Box was designed to facilitate implementation of recommendations. This guideline will be distributed widely through the ASCO Practice Guideline Implementation Network. ASCO resource-stratified guidelines are posted on the ASCO Web site and most often published in JGO and *Journal of Oncology Practice*. Specific ideas around the implementation plan include:

1. Dissemination through international palliative care organizations, including the International Association for Hospice and Palliative Care and multilaterals, eg, WHO
2. Working with local/regional oncology societies, including societies with whom Panel members and/or ASCO have relationships (eg, African Organisation for Research and Treatment in Cancer, African Palliative Care Organization, Asia Pacific Hospice Network, African Palliative Care Association, Asociación Latinoamericana de Cuidados Paliativos, European Association for Palliative Care).
3. Patient advocacy groups/NGOs/civil societies, eg, European Coalition of Cancer Patients (and many others)
4. ASCO Communications and ASCO International (will likely include asco.org, media outreach, including to international reporters, ASCO e-mails/news releases, www.cancer.net, ASCO Connection [member magazine], social media, other member communications; may include ASCO International courses/workshops, ASCO University, etc, depending on those programs’ needs)
5. Conferences—potentially ASCO Annual Meeting, AAHPM/ASCO/American Society for Radiation Oncology(ASTRO)/Multinational Assocation of Supportive Care in Cancer (MASCC) Palliative Care Symposium, and AORTIC
6. Non-ASCO journals’ news briefs, eg, *Journal of Palliative Medicine*, *Journal of Pain and Symptom Management*

## ADDITIONAL RESOURCES

More information, including a Data Supplement with additional evidence tables, a Methodology Supplement with information about evidence quality and strength of recommendations, slide sets, and clinical tools and resources, is available at www.asco.org/resource-stratified-guidelines. Patient information is available at www.cancer.net. 

Related ASCO Guidelines

- Integration of Palliative Care into Standard Oncology Practice^1^ (http://ascopubs.org/doi/10.1200/JOP.2016.017897)
- Management and Care of Women With Invasive Cervical Cancer Resource-Stratified Guideline^70^ (http://ascopubs.org/doi/10.1200/JGO.2016.003954)
- Patient-Clinician Communication^71^ (http://ascopubs.org/doi/10.1200/JCO.2017.75.2311)
- Management of Chronic Pain in Survivors of Adult Cancers^5^ (http://ascopubs.org/doi/10.1200/JCO.2016.68.5206)
- Other ASCO Supportive Care and Treatment-Related Issue Guidelines: www.asco.org/supportive-care-guidelines

ASCO believes that cancer clinical trials are vital to inform medical decisions and improve cancer care and that all patients should have the opportunity to participate. Patients in clinical trials may benefit from the support of palliative care.

## Figures and Tables

**Table A1:**
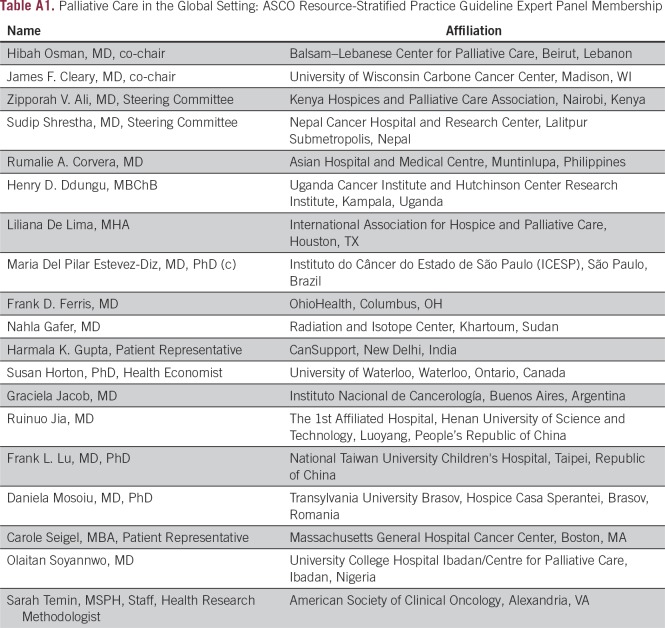
Palliative Care in the Global Setting: ASCO Resource-Stratified Practice Guideline Expert Panel Membership
